# Thrombotic Thrombocytopenic Purpura Associated with Mixed Connective Tissue Disease: A Case Report

**DOI:** 10.1155/2011/953890

**Published:** 2011-09-11

**Authors:** João Tadeu Damian Souto Filho, Philipe Vianna de Barros, Aline Maria Yamaguti Rios Paes da Silva, Fernanda Alves Barbosa, Gustavo Fernandes Ribas

**Affiliations:** ^1^Faculdade de Medicina de Campos, 28035-580, Campos dos Goytacazes, RJ, Brazil; ^2^Hospital Ferreira Machado and Hospital Geral de Guarus, Campos dos Goytacazes, RJ, Brazil

## Abstract

Thrombotic thrombocytopenic purpura (TTP) is a multisystemic disorder characterized by microangiopathic hemolytic anemia and thrombocytopenia, which may be accompanied by fever, renal, or neurologic abnormalities. Cases are divided into acute idiopathic TTP and secondary TTP. Autoimmune diseases, especially systemic lupus erythematosus, in association with TTP have been described so far in many patients. In contrast, TTP occurring in a patient with mixed connected tissue disease (MCTD) is extremely rare and has only been described in nine patients. We describe the case of a 42-year-old female with MCTD who developed thrombocytopenia, microangiopathic hemolytic anemia, fever, and neurological symptoms. The patient had a good clinical evolution with infusion of high volume of fresh frozen plasma, steroid therapy, and support in an intensive care unit. Although the occurrence of TTP is rare in MCTD patients, it is important to recognize TTP as a cause of thrombocytopenia and hemolytic anemia in any patient with autoimmune diseases. Prompt institution of treatment remains the cornerstone of treatment of TTP even if plasma exchange is not available like what frequently happens in developing countries.

## 1. Introduction

Thrombotic thrombocytopenic purpura (TTP) is a multisystemic disorder characterized by microangiopathic hemolytic anemia and thrombocytopenia, which may be accompanied by fever, renal, or neurologic abnormalities [1, 2]. It is almost always acquired, with rare cases of congenital TTP (Upshaw-Schulman syndrome). Acquired cases are divided into acute idiopathic TTP and secondary TTP, which has been seen in association with collagen vascular disease, bone marrow transplantation, malignancy, pregnancy, infections, and drugs such as cyclosporine, tacrolimus, ticlopidine, and antineoplastic agents [1, 3]. Systemic lupus erythematosus (SLE), antiphospholipid antibody syndrome, adult onset Still's disease, systemic sclerosis, polymyositis, dermatomyositis, and rheumatoid arthritis in association with TTP have been described so far in many patients [4–12]. In contrast, mixed connected tissue disease (MCTD) in association with TTP is extremely rare and has only been described in nine patients [13–21]. Here, we describe the 10th case of mixed connective tissue disease (MCTD) complicated by TTP and discuss the complexity of its management in a developing country.

## 2. Case Report

A 42-year-old Afro-Brazilian woman was admitted to emergency department with gradual neurologic disorientation, associated with severe headache and episodes of vomiting for three days. The patient had a past history of mixed connective tissue disease (MCTD) for 4 years. Her clinical presentation included Raynaud's phenomenon associated with puffy fingers, arthralgias, mild arthritis (nonerosive and nondeforming), and myalgia. She also had a pericardial effusion 3 years ago that seems to be related to MCTD. Laboratory studies revealed that the patient had an ESR of 70 mm/hr, positive rheumatoid factor (titre >128), positive ANA (1 : 1280) with speckled pattern, and positive anti-U1-RNP (>240 U/mL). Anti-DNA, Anti-SM, Anti-CCP, Anti-Jo1, Anti-SS-A, Anti-SS-B, Anti-SCL-70, Anticentromere, ANCA, and Anticardiolipin were negative. She reported having used only prednisone for the treatment of MCTD, currently using 10mg/kg/day. She also had hypertension on regular treatment for the last 10 years.

**Figure 1:**
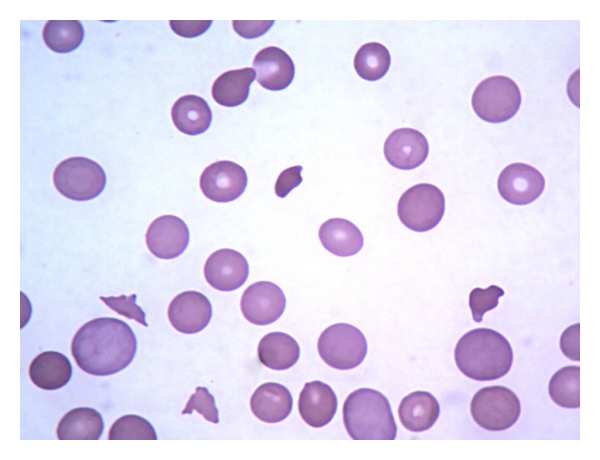
Blood Smear with anisocytosis, poikilocytosis, polychromatophilia (polychromasia) with schistocytes, and thrombocytopenia (1000x).

On admission she was awake, dehydrated (+/4+), pale (2+/4+), confused, and disorientated with a Glasgow coma scale of 13 points and without focal neurologic deficit. The blood pressure was 180/100 mmHg, pulse 108 per min. and temperature 37.2°C. Clinical examinations of the cardiovascular, respiratory systems, and abdomen were normal. There was edema in the lower limbs (2+/4+). Complete blood cell count showed a normocytic normochromic anemia and thrombocytopenia (Table 1). Peripheral blood smear demonstrated anisocytosis, poikilocytosis with schistocytes, and thrombocytopenia (Figure 1). Biochemical analysis revealed elevated levels of serum LDH (1,830 U/L) and bilirubin (total 4.1 mg/dL, direct 2.1 mg/dL and indirect 2.0 mg/dL). Direct Coombs test was negative, and PT and aPTT were normal. Her urine exam was positive to protein (3+), leukocyte (2+), hemoglobin, (4+), bilirubin (1+), and urobilinogen (2+) with negative nitrite. Other laboratory data are summarized in Table 1.

Clinical identification of nonimmune haemolytic anemia with presence of red cell fragmentation, thrombocytopenia, and altered level of consciousness led to the diagnosis of thrombotic thrombocytopenic purpura secondary to autoimmune disease, since the patient was previously diagnosed as MCTD. Prednisone 1 mg/kg/day (80 mg/day) scheme was initiated after administration of antiparasitic therapy (prophylaxis of strongyloides hyperinfection syndrome [22]), fresh frozen plasma (15 mL/kg/day), and red blood cells transfusion with blood pressure monitoring and use of diuretics to prevent fluid overload.

She evolved in 24 hours with high fever (39.2°C); blood pressure was 160/80 mmHg, and subsequently, with decreased level of consciousness, she underwent endotracheal intubation and was transferred to the Intensive care unit (ICU). The infusion of fresh frozen plasma was progressively increased to 25–30 mL/kg/day. During hospitalization, she presented with pneumonia and received ceftriaxone and clindamycin. Abdominal ultrasonography, computed tomography, and magnetic resonance imaging of the brain showed no specific abnormalities.

After 5 days in the ICU, she showed good clinical outcome, with improvement of neurologic and respiratory parameters, allowing weaning and withdraw of mechanical ventilation. Throughout the period her blood pressure was kept under strict control with captopril, amlodipine, atenolol, methyldopa, and losartan, as well as periods of sodium nitroprusside.

After 20 days of hospitalization, the patient presented progressive improvement in microangiopathic anemia, increased haemoglobin, and platelets, and a progressive decrease of LDH and reticulocytes (Table 1). She was not submitted to plasmapheresis. There was normalization of blood pressure with concomitant gradual reduction in the volume of fresh frozen plasma infused and reduction of prednisone. She was discharged from the hospital with antihypertensive drugs (clonidine and amlodipine) and prednisone (20 mg/day). After 40 days of symptom onset and 20 days after discharge the patient was asymptomatic and laboratory tests improved (Table 1).

## 3. Discussion

Following the discovery that TTP is associated with a severe deficiency of ADAMTS13 activity [23, 24], it was suggested that ADAMTS13 deficiency may become the definition and diagnostic criterion for TTP. But many patients who fulfill the clinical diagnostic criteria for TTP do not have ADAMTS13 deficiency [10, 25]. For some authors, measurements of ADAMTS13 activity are not required and do not conclusively confirm the diagnosis of TTP [26]. In this way, the diagnosis of TTP is based on the presenting clinical features [1]. Prompt diagnosis of TTP is critical to begin treatment and reduce patient's mortality.

Patients diagnosed with TTP may have additional disorders including autoimmune diseases. In this group systemic lupus erythematosus (SLE) is the most common, followed by antiphospholipid antibody syndrome, adult onset Still's disease, rheumatoid arthritis, systemic sclerosis, polymyositis and dermatomyositis [4–12].

TTP associated with mixed connected tissue disease is rare and has only been described in nine patients (Table 2) [13–21]. Most of these cases were women (8 : 1) with the median age of 40 years. The mortality was high (45%), early, (within 2–45 days) and associated with serious neurological impairment (seizure and coma). Our case presented a good clinical evolution in spite of serious neurological manifestations which points to a worse prognosis [17, 19].

The pathogenic processes of thrombotic microangiopathy in patients with connective tissue diseases are heterogeneous. ADAMTS13 activity is significantly decreased in this group of patients. However, only a minor population presents neutralising autoantibodies against ADAMTS13, which was associated with severe ADAMTS13 deficiency, lower platelet counts, and better therapeutic outcomes. A major population has normal or moderately reduced ADAMTS13 activity [4]. In this group, the mechanisms of development of TTP remain unclear. There is a higher prevalence of anti-endothelial cell antibodies in the sera of MCTD patients [27] and depressed plasma fibrinolytic activity [28], suggesting that a MCTD related vasculitis and thrombotic microangiopathy may have been involved in the pathogenesis of TTP [15, 17]. In patients with systemic sclerosis, renal crisis remains one of the potential processes related to microangiopathic hemolytic anemia, thrombocytopenia, accelerated hypertension and acute renal failure [29, 30].

Previous reported cases were treated with various therapies including steroids, fresh frozen plasma (FFP), plasma exchange (PE), prostacyclin, vincristine, cyclosporine, cyclophosphamide, rituximab and aspirin [13–21]. Today, the mainstay of treatment of acute TTP is plasma exchange [1]. Before the plasma treatment era, survival of patients with TTP was only 10%. Then, almost 20 years ago, plasma exchange was reported to increase survival to 78%, compared to 51% survival for patients treated with plasma infusion [31]. Nowadays, however, there are some places that plasma exchange still unavailable. In these cases, high volume of plasma infusion (30 mL/kg/d) may be indicated if there is to be an unavoidable delay in plasma exchange [1, 32, 33]. Adjuvant corticosteroid treatment is also used with the intention to suppress the activity of auto-antibodies against ADAMTS13 [1, 26].

The clinical team's intention was to transfer the patient to start plasmapheresis, but by the critical condition, it was not possible immediately. So, we started FFP infusion and immunosuppression with prednisone 1 mg/kg/day and the patient gradually improved. The high volume of fresh frozen plasma could only be provided because of patient's good cardiac and renal function associated with a strict blood pressure control.

## 4. Conclusion

Although the occurrence of TTP is rare in MCTD patients, it is important to recognize TTP as a cause of thrombocytopenia and hemolytic anemia in any patient with autoimmune diseases. Prompt institution of treatment remains the cornerstone of treatment of TTP even if plasma exchange is not available like what frequently happens in developing countries.

## Figures and Tables

**Table 1:** Laboratory data.

	On admission	After 20 days	After 40 days
Complete blood count			
RBC (×10^12^/L)	2.88	3.97	4.55
Hemoglobin (g/dL)	7.8	11.5	12.4
Hematocrit (%)	23.8	35	39.6
MCV (fL)	82.6	88	87
MCH (pg)	27	29	27
MCHC (g/dL)	32.7	33	31
RDW (%)	21.2	17.6	13.2
WBC (×10^9^/L)	9.8	5.72	7.23
Platelet (×10^9^/L)	5.1	243	312
Reticulocytes (%)	8.4	1.1	0.8

Blood chemistry			
AST (IU/L)	46	22	21
ALT (IU/L)	43	29.2	31
GGT (IU/L)	98	230	80
ALP (IU/L)	98	130	72
LDH (IU/L)	1830	365	176
Total bilirubin (mg/dL)	4.1	0.5	0.6
Direct bilirubin (mg/dL)	2.1	0.23	0.28
Indirect bilirubin (mg/dL)	2	0.27	0.32
Glucose (mg/dL)	128	98	89
Urea (mg/dL)	19		
Creatinine (mg/dL)	0.6		
Uric acid (mg/dL)	3.0		
Sodium (mEq/L)	139		
Potassium (mEq/L)	2.9		

Hormones			
TSH (*μ*U/mL)	2.72		
Free T4 (pg/dL)	1.08		

Serology			
HBsAg	Negative		
Anti-HBs	Negative		
Anti-HBc	Negative		
Anti-HCV	Negative		
Anti-HIV	Negative		
Blood culture	Negative		

Other blood tests			
ESR (mm/hr)	78		
PT (s)	10.2		
aPTT (s)	21.3		
Direct Coobs test	Negative		

Urinalysis			
Protein	(3+)		
Leukocyte	(2+)		
Hemoglobin	(4+)		
Bilirubin	(1+)		
Urobilinogen	(2+)		
Nitrite	(—)		
Urine culture	negative		

RBC: red blood cells, MCV: mean corpuscular volume, MCH: mean corpuscular hemoglobin, MCHC: mean corpuscular hemoglobin concentration, RDW: red blood cell distribution width, WBC: white blood cells, AST: aspartate aminotransferase, ALT: alanine aminotransferase, ALP: alkaline phosphatase, GGT: gamaglutamil transferase, LDH: lactate dehydrogenase, ESR: erythrocyte sedimentation rate, PT: prothrombin time, ATTP: activated partial thromboplastin time, TSH: Thyroid-stimulating hormone.

**Table 2:** Characteristics of TTP in patients with MCTD.

Age	Sex	Duration of MTCD(years)	Prodrome	Treatment	Prognosis	Reference
29	F	5	Chest pain, fever, loin pain, confusion	PDN	Died (2 days)	[13]
33	F	2	Flu-like syndrome	PDN, FFP, vincristin, prostacyclin	Alive	[14]
40	F	2	Headache, confusion, seizure	PE, PDN, vincristin, aspirin	Died (12 days)	[15]
55	F	?	Fever, confusion, seizure, coma, myocardial infarction	PE, PDN	Alive	[16]
64	F	8	Headache, confusion seizure, coma	PE, PDN	Died (45 days)	[17]
15	M	?	Headache, visual blurring, vomiting, Paresthesia	PE, PDN, vincristin, cyclosporine	Alive	[18]
73	F	10	Confusion, coma	PE, PDN	Died (34 days)	[19]
46	F	2	Fever, headache	PE, PDN, aspirin, Cyclophosphamide, rituximab	Alive	[20]
24	F	<1	Laboratory detection	PE	Alive	[21]
42	F	4	Headache, confusion seizure, coma, Fever, vomiting, hematuria	FFP, PDN	Alive	This case

F: female, M: male, PDN: prednisone or prednisolone, FFP: fresh frozenplasma, PE: plasma exchange.
